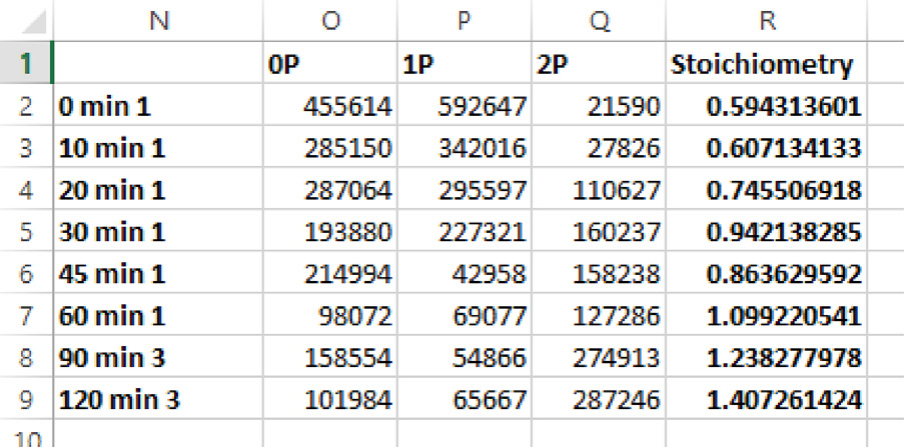# Tools and protocol for quantification of myosin phosphorylation with MRM-MS

**DOI:** 10.1016/j.mex.2018.05.004

**Published:** 2018-05-17

**Authors:** Justin A. MacDonald, Annegret Ulke-Lemée, Mona Chappellaz, Hayden Segboer

**Affiliations:** Department of Biochemistry & Molecular Biology, Cumming School of Medicine, University of Calgary, Calgary, AB, T2N 4Z6, Canada

**Keywords:** MRM-MS myosin phosphorylation assay, Smooth muscle, Myosin regulatory light chain, LC20, MLC, Artery, Intestine, Targeted proteomics, Quantitation, Stoichiometry, Phosphorylation, Serine 19, Threonine 18

## Abstract

The phosphorylation of myosin regulatory light chain (LC20) at Thr18 and Ser19 is positively correlated with tension development in smooth muscle tissue, and the molar stoichiometry of LC20 phosphorylation is commonly profiled as a measure of smooth muscle contractility. We provide details for a newly applied multiple reaction monitoring (MRM)-mass spectrometry (MS) method for the quantification of LC20 phosphorylation at Thr18 and Ser19. This MRM-MS method provides a robust alternative to antibody-based detection systems (such as Phos-Tag SDS-PAGE) for the quantification of LC20 phosphorylation.

## Highlights

• The MRM-MS assay performs equally to Phos-tag SDS-PAGE for the determination of LC20 phosphorylation.
• Eliminates ambiguities in determinations of LC20 phosphorylation that may arise from western blotting.
• Can precisely define the molar stoichiometry of Thr18 and Ser19 phosphorylation.

## Graphical abstract

**Figure:**
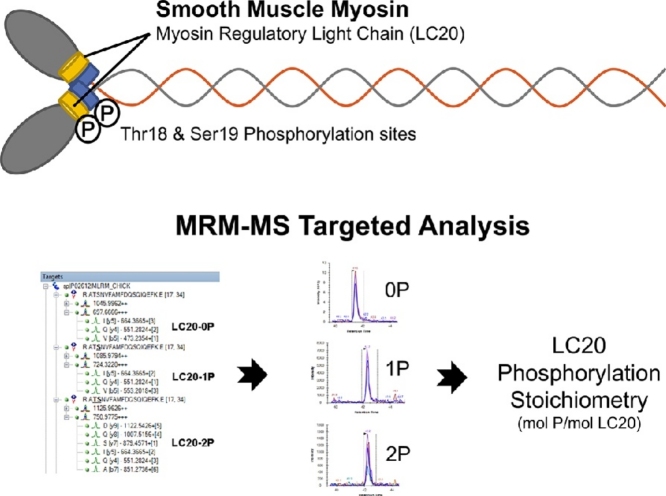

**Specifications Table**

**Table:** 

Subject area	Biochemistry, Genetics and Molecular Biology
More specific subject area	Targeted proteomics
Method name	MRM-MS Myosin Phosphorylation Assay
Name and reference of original method	Not applicable
Resource availability	Skyline v3.7 program – https://skyline.ms/ Skyline Panorama Public Repository – https://panoramaweb.org

## Materials

### Skyline software for targeted mass spectrometry environment

• Skyline is a freely-available, open-source Windows client application for building targeted quantitative proteomic methods and analyzing the resulting mass spectrometry data [1,2]. The Skyline v3.7 program is available for download from the MacCross Laboratory website: https://skyline.ms/project/home/software/Skyline/begin.view
• Panorama open-source repository server application for targeted proteomic assays that integrate into Skyline MRM-MS workflows [3]. Panorama is also available via the MacCross Laboratory website: https://panoramaweb.org.

### *In vitro* generation of phosphorylated LC20 protein

• Smooth muscle myosin light chain (LC20), myosin light chain kinase (MLCK), and calmodulin proteins were purified from chicken gizzard as previously described ([[4], [5], [6]], respectively).
• HEPES buffer: 4-(2-hydroxyethyl)-1-piperazine ethanesulfonic acid (200 mM) Dissolve in dH_2_O and adjust pH to 7.4.
• MgCl_2_ (100 mM) and CaCl_2_ solutions (100 mM). Dissolve in dH_2_O.
• ATP: adenosine 5’-triphosphate (100 mM). Dissolve in Tris-(hydroxymethyl) aminomethane hydrochloride (TRIS-HCl, 25 mM, pH 8.0). Neutral ATP solutions stored frozen at −20 °C are stable for at least one year.
• EDTA/EGTA quenching buffer: prepared by mixing 336 μl of 0.2 M EDTA and 192 μl of 0.2 M EGTA to give a final stock solution of 126 mM EDTA & 73 mM EGTA.

### Preparation of smooth muscle tissue extracts

• TCA/DTT/acetone: 10% (w/v) trichloroacetic acid, 10 mM dithiothreitol in ice cold acetone.
• DTT/acetone: 10 mM dithiothreitol in ice cold acetone
• Lyophiliser- Freeze Dry System (Labconco, Freezone 6)
• Tissue extraction buffer: 50 mM ABC, pH 8.2, 50 mM NaCl, 1 M urea, 2% (w/v) sodium deoxycholate, 1 mM DTT and cOmplete protease inhibitor cocktail (Millipore-Sigma). LC20 is primarily associated with the insoluble fraction during isolation of muscle proteins with centrifugation, so we include of 2% (w/v) sodium deoxycholate and 1 M urea to enhance the solubilization efficiency.
• Micro ground-glass, Potter-Elvehjem tissue homogenizer
• Vortex shaker
• Refrigerated micro-centrifuge

### Tryptic digestion

• ABC: 50 mM ammonium bicarbonate, pH 8. Make fresh by dissolving 0.039 g ammonium bicarbonate in 10 ml dH_2_O. The ABC solution should have a pH of approximately 8. The pH does not need to be further adjusted.
• BSA: 10 mg/ml bovine serum albumin. Make fresh by dissolving in 50 mM ABC.
• IAA: 200 mM stock iodacetamide. Make fresh by dissolving 0.037 g iodoacetamide in 1 ml of 50 mM ABC.
• DTT: 1 M stock dithiothreitol, dissolve 0.154 g in 1 ml pure H_2_O. Stored at −80 °C in small aliquots. Discard and do not refreeze once thawed. The DTT is not soluble in ice-cold ABC, and the solution must be warmed sufficiently to fully dissolve DTT.
• Mass spectrometry grade trypsin: 0.5 mg/ml (Promega). Solution made according to the manufacturer’s manual. In brief, add 40 μl of the supplied resuspension buffer to one vial of trypsin, swirl the vial to distribute the buffer, do not vortex. The glass vial can be centrifuged gently (5 min at 500 x *g*) by placing it into a 50 ml Falcon tube that has been cushioned with laboratory wipes. We typically obtain ∼39 μl from a 40 μl original volume. Store according to manufacturer’s suggestions.
• Heating block for 1.5 ml tubes. Constant temperature settings of 50 °C and 37 °C.
• Microcentrifuge for 1.5 ml tubes, capable of reaching >12,000 rpm.
• TFA, trifluoroacetic acid, HPLC-grade.
• ACN, acetonitrile, HPLC-grade.
• TFA/ACN solution: 0.5% (v/v) TFA/50% (v/v) ACN; make up fresh by mixing 10 μl TFA, 100 μl ACN and 90 μl pure H_2_O.
• Autosample vials (*e.g.*, 300 μl polypropylene vials with snap lids)

### Profiling LC20 phosphorylation with MRM-MS

• HPLC: Dionex Ultimate 3000, running Chromeleon Express and Dionex Chromatography MS Link or similar HPLC system.
  Trap column – for peptide concentration and desalting with capacity for up to 200 μg peptide (*e.g.* OPTI-TRAP Macro column – Peptide 50 μl, large capacity, 3 mm × 12 mm; Optimize Technologies).
  Precolumn – same bead type and same or smaller size as the separation column connected just upstream of the separation column (*e.g.,* C18 PepMap column – 100 Å pore size, 5 μm particle size, 5 mm length, 300 μm i.d.; ThermoFisher Scientific).
  Separation column – for providing analytical separation of tryptic peptides. (*e.g.*, PepMap300 C18 column, 1 × 150 mm, C18,  μm particle size, 300 Å pore size; ThermoFisher Scientific).
  Organic running buffer – HPLC-grade ACN with 0.5% (v/v) formic acid.
  Aqueous running buffer – HPLC-grade H_2_O with 0.5% (v/v) formic acid.
• Mass spectrometer: QTRAP (ABSciex QTrap4500, running Analyst software) or similar.
  Instrument settings - TurboSpray Ion Source, 5500 V ion spray voltage (IS) in positive mode, 25 curtain gas (CUR), 20 V ion source gas 1 (GS1), and 25 V ion source gas 2 (GS2).

## Methods

### Preparation of phosphorylated LC20 protein standards

• Phosphorylated LC20 can be generated *in vitro* by reaction with MLCK [7]. The components of the kinase reaction are provided in Table 1. Monophosphorylation of S19 is promoted with low CaM-MLCK content and short reaction duration (<5 min) while diphosphorylation of T18 and S19 is induced with high CaM-MLCK content and long reaction duration (45–60 min). Reactions are initiated by the sequential addition of MgCl_2_ – ATP solution and then MLCK, vortexed gently to ensure complete mixing of constituents, incubated at 30 °C, and terminated by addition of EDTA/EGTA quenching buffer (*i.e.*, addition of 21.6 μl of 126 mM EDTA, 73 mM EGTA solution).

**Table 1:** Reaction components for developing LC20 Phosphorylation.

	LC20-1P (pS19)	LC20-2P (pT18pS19)
HEPES, pH 7.4 (200 mM)	25 mM	25 mM
CaCl_2_ (100 mM)	0.1 mM	0.1 mM
LC20 (4 mg/ml)	20 μg	20 μg
calmodulin (5 mg/ml)	10 μg/ml	20 μg/ml

ATP (100 mM)	0.2 mM	0.2 mM
MgCl_2_ (100 mM)	2 mM	2 mM

MLCK (1 mg/ml)	1 μg/ml	60 μg/ml
Final Reaction Volume:	300 μl	300 μl
Reaction Duration:	1–5 min	45–60 min

### Preparation of smooth muscle tissue extracts

• The extraction of proteins from smooth muscle tissue is most effective following lyophilisation (>16 h). Smooth muscle tissue can be quenched by immersion in 10% (w/v) TCA, 10 mM DTT in acetone (ice-cold). This procedure inactivates protein kinase and protein phosphatase activities and preserves LC20 phosphorylation status. The lyophilised smooth muscle tissues are typically stored at −80 °C so that multiple myography samples can be analyzed with batch processing.
• Remove samples from −80 °C storage and keep on ice.
• Immediately add ice-cold extraction buffer containing 50 mM AMC, pH 8.2, 50 mM NaCl, 1 M urea, 2% (w/v) sodium deoxycholate, 1 mM DTT and Complete protease inhibitor cocktail. Add 0.25 ml for rat caudal artery strips (∼6 mg tissue) and 0.1 ml for rat ileal smooth muscle strips (∼2.5 mg tissue).
• Homogenize tissue with a micro Potter-Elvehjem homogenizer. Take care to ensure the small tissue strip remains immersed in extraction buffer until it is completely solubilized. Transfer tissue extract to a 0.5 ml microcentrifuge tube.
• Vortex the tissue homogenate for 2 h at 5 °C.
• Clarify the extract by centrifugation – 14,000 rpm, 10 min, 5 °C.
• Quantify protein concentration of tissue extract by BCA.

### Tryptic digestion of protein samples

A Purified LC20 protein: Combine LC20 protein (1 μg) with BSA carrier protein (9 μg; 0.9 μl of a 10 mg/ml solution) and dilute with 50 mM ABC to a final volume of 54 μl.
B Tissue extract: Combine total protein (2 μg) with BSA carrier protein (8 μg; 0.8 μl of a 10 mg/ml solution) and dilute with 50 mM ABC to a final volume of 54 μl.
C Complete protein reduction of the sample with the addition of DTT (0.3 μl from a 1 M stock to give 5 mM final concentration) and incubate for 30 min at 50 °C.
D Complete alkylation of the sample with the addition of iodoacetamide (4.6 μl of 200 mM solution to give a final concentration of 15 mM). Incubate for 30 min in the dark at room temperature.
E Quench any residual iodoacetamide with a second addition of DTT (0.3 μl from a 1 M stock) and incubation for 30 min at 50 °C.
F Digest protein with trypsin (2.5 μl of 0.5 μg/μl trypsin solution) for 16 h at 37 °C. The incubation can be completed in a thermoblock or PCR instrument to maintain consistent temperature throughout. Following the incubation, centrifuge the samples to collect all liquid at the bottom of the tubes.
G The tryptic peptide solution is acidified. Add 6 μl of TFA/ACN solution (5% TFA (v/v), 50% (v/v) ACN in H_2_O). Mix thoroughly and then centrifuge (12,000 rpm, 10 min, 22 °C) to remove particulate matter. For tissue extraction samples, this step will precipitate deoxycholate. Carefully transfer the clarified supernatant to a new tube.
H Repeat the centrifugation step and transfer the peptide mixture to HPLC-autosampler vials. Tryptic peptide purification using C18 ZIP-TIPs prior to HPLC can be omitted if the user prefers. Using a trap-column in the HPLC method achieves the same peptide purification and removal of unwanted contaminants without additional processing steps.

### Skyline analysis criteria for MRM-MS

• Create an Analyst program using the transition list from Table 2 by copy-pasting the transitions from a Microsoft Excel worksheet into a new MRM-Analyst method. Ensure that “MRM Method” and “Scheduling” is selected in Analyst and that the methods table is the same format as the transition list.

**Table 2:** List of Precursor and Transitions Used in the MRM-MS Assay.

Q1	Q3	RT	Sequence	DP	EP	CE	CXP
623.796	–		LC20-pan,GNFNYVEFTR,+2	–	–	–	–
623.796	552.28		LC20-pan,EFTR,+1y4	76.6		28.0	
623.796	651.35		LC20-pan,VEFTR,+1y5	76.6		28.0	
623.796	928.45		LC20-pan,NYVEFTR,+1y7	76.6		28.0	
697.667	–		LC20-0P,ATSNVFAMFDQSQIQEFK,+3	–	–	–	–
697.667	551.28		LC20-0P,QEFK,+1y4	82.0		25.6	
697.667	664.37		LC20-0P,IQEFK,+1y5	82.0		27.6	
697.667	473.24		LC20-0P,ATSNV,+1b5	82.0		23.6	
724.322	–		LC20-1P,AT[pS]NVFAMFDQSQIQEFK,+3				
724.322	551.28		LC20-1P,QEFK,+1y4	83.9		27	
724.322	553.20		LC20-1P,AT[pS]NV,+1b5[P]	83.9		25	
724.322	664.36		LC20-1P,IQEFK,+1y5	83.9		29	
750.978	–		LC20-2P,A[pT][pS]NVFAMFDQSQIQEFK,+3	–	–	–	–
750.978	551.28		LC20-2P,QEFK,+1y4	85.9		26.3	
750.978	879.46		LC20-2P,SQIQEFK,+1y7	85.9		30.3	
750.978	851.27		LC20-2P,A[pT][pS]NVFA,+1b7[2P]	85.9		36.3
• Select an MRM detection window of 10 min. The retention time predictor in Skyline estimates retention times with a certain error due to the inherent inaccuracy of the method; thus, we initially select a wide retention time window to ensure all peptides are detected. The detection window can then be narrowed for future analyses, if desired. Some retention time drift across many sample injections is possible and can indicate issues with the HPLC (*e.g.*, variability in pressure and/or flow). Retention time drift will also occur with replacement of HPLC plumbing and/or columns. An altered retention time for a peptide often introduces interferences (false positives). In this case, the peaks need to be critically examined.

Table of peptides and their respective ions as entered into Analyst. The table is generated by Skyline and edited in Microsoft Excel. Abbreviations are: Q1, Precursor mass (*m/z*); Q3, Fragment mass (*m/z*), RT, retention time in min; DP, declustering potential; EP, Entrance potential; CE, Collision energy; and CXP, Cell exit potential. The tryptic peptide sequences are provided along with associated transition (y-ion or b-ion) and charge state (+2 or +3). [pS] and [pT] indicate a phosphorylated residue.

• Create a “Dionex Chromatography MS Link” HPLC program.
• The HPLC is set up with a trap column under reverse elution conditions. This ensures that the injected peptides are sequestered onto the trap column, washed, then reverse-eluted onto the separation column during the ensuing gradient.
• Set an HPLC program with a flow rate of 50 μl/min and a gradient from 2 to 50% (v/v) ACN with 0.1% (v/v) formic acid and eluting peptides analysed in-line by the MS.
• Use the partial inject mode with 15 μl injection volume.
• Once an Analyst and HPLC program is created, load the samples into the autosampler and create a batch to submit the samples. Ensure there are no air bubbles in the sample tubes. As a first sample, inject 5% ACN, 0.5% TFA to equilibrate the system, using the same HPLC method. If a positive control is used (*i.e.*, phosphorylated LC20 protein), this sample should be loaded last to avoid possible bleed-through into subsequent runs.

### Data analysis

• To quantify the molar stoichiometry of LC20 phosphorylation with MRM-MS, the peak areas of selected transitions for [LC20-0P], [LC20-1P] and [LC20-2P] (provided in Table 2) were defined using Skyline with automatic background subtraction.

### Calculation of LC20 phosphorylation stoichiometry

• The molar stoichiometry of LC20 phosphorylation was calculated as: mol phosphate (P)/mol LC20 = ([LC20-1P peak area] + (2 × [LC20-2P peak area]))/([LC20-0P peak area] + [LC20-1P peak area] + [LC20-2P peak area]).

### Method validation

• The MRM-MS method was previously assessed in a head-to-head comparison with the Phos-tag SDS-PAGE method for determination of LC20 phosphorylation stoichiometry [7]. Results acquired with the two techniques indicate that the MRM-MS assay performs equally to Phos-tag SDS-PAGE for the accurate determination of LC20 phosphorylation stoichiometry.
• As an example of the MRM-MS analysis steps employed in the method, a batch of eight rat caudal artery strips, that had been experimentally treated with calyculin A (0.5 μM, 0–120 min) to induce LC20 phosphorylation and contractile force development, were examined.
• As detailed in Section Skyline analysis criteria for MRM-MS above, Q1 precursor and Q3 transition masses for the LC20 peptides were entered into the Analyst program. Also included were empirically determined mass spectrometer conditions for predicted elution time (a 10 min window provided), declustering potential (DP), entrance potential (EP), cell exit potential (CXP) and collision energy (CE).

**Figure:**
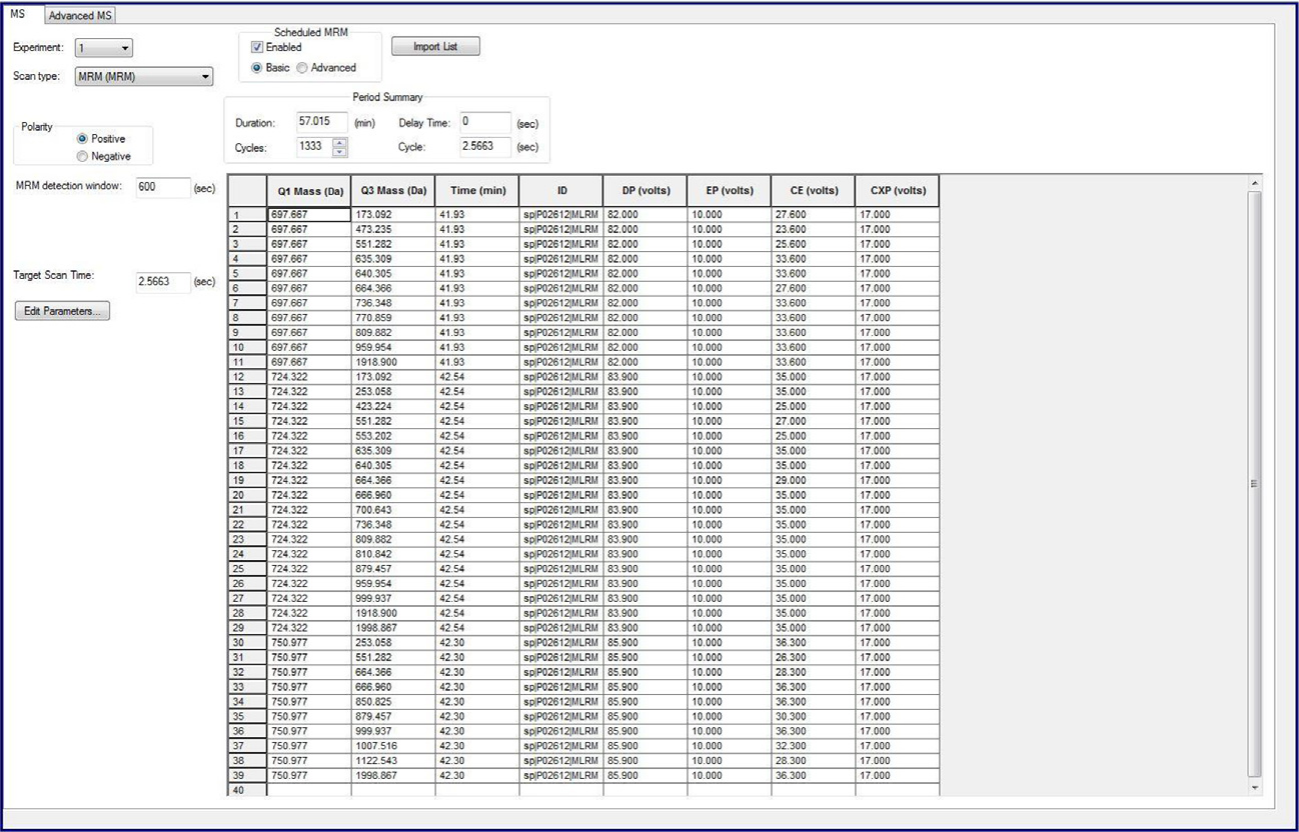

• Next, the data collected during the MRM-MS run was accessed in the Skyline program by up-loading the Analyst “*.wiff” file. A screen capture from Skyline is provided below and shows the LC20-0P, LC20-1P and LC20-2P precursor peptides and transitions used to generate LC20 phosphorylation stoichiometry. Also provided are the precursor and transitions used for a pan-LC20 peptide (GNFNYVEFTR) located in the C-terminus of the LC20 protein.

**Figure:**
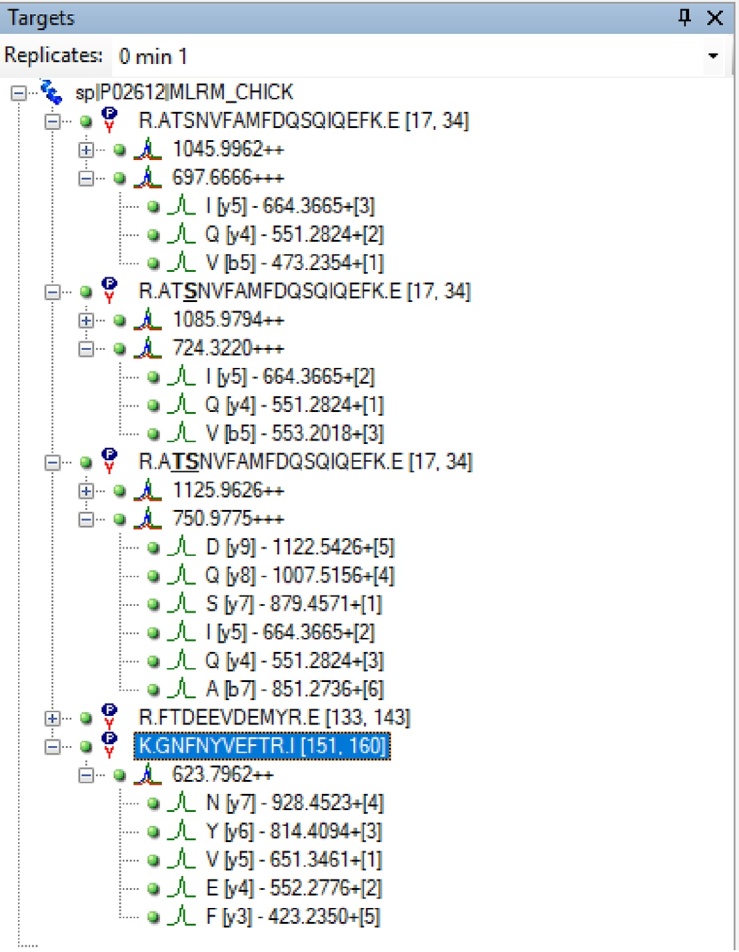

• A representative elution profile for the eight different tissue samples is shown for each of the LC20-0P, LC20-1P and LC20-2P precursor peptides and transitions obtained with the MRM-MS method. Retention times for the peptide elutions are provided in the lower left panels. The peak areas for the MRM-MS parameters are provided in the low right panel of each image. Note the relative decline in LC20-0P signals and the relative increase in LC20-2P signals with longer exposure of arterial tissue to calyculin A.
  **LC20-0P**
  **Figure:**
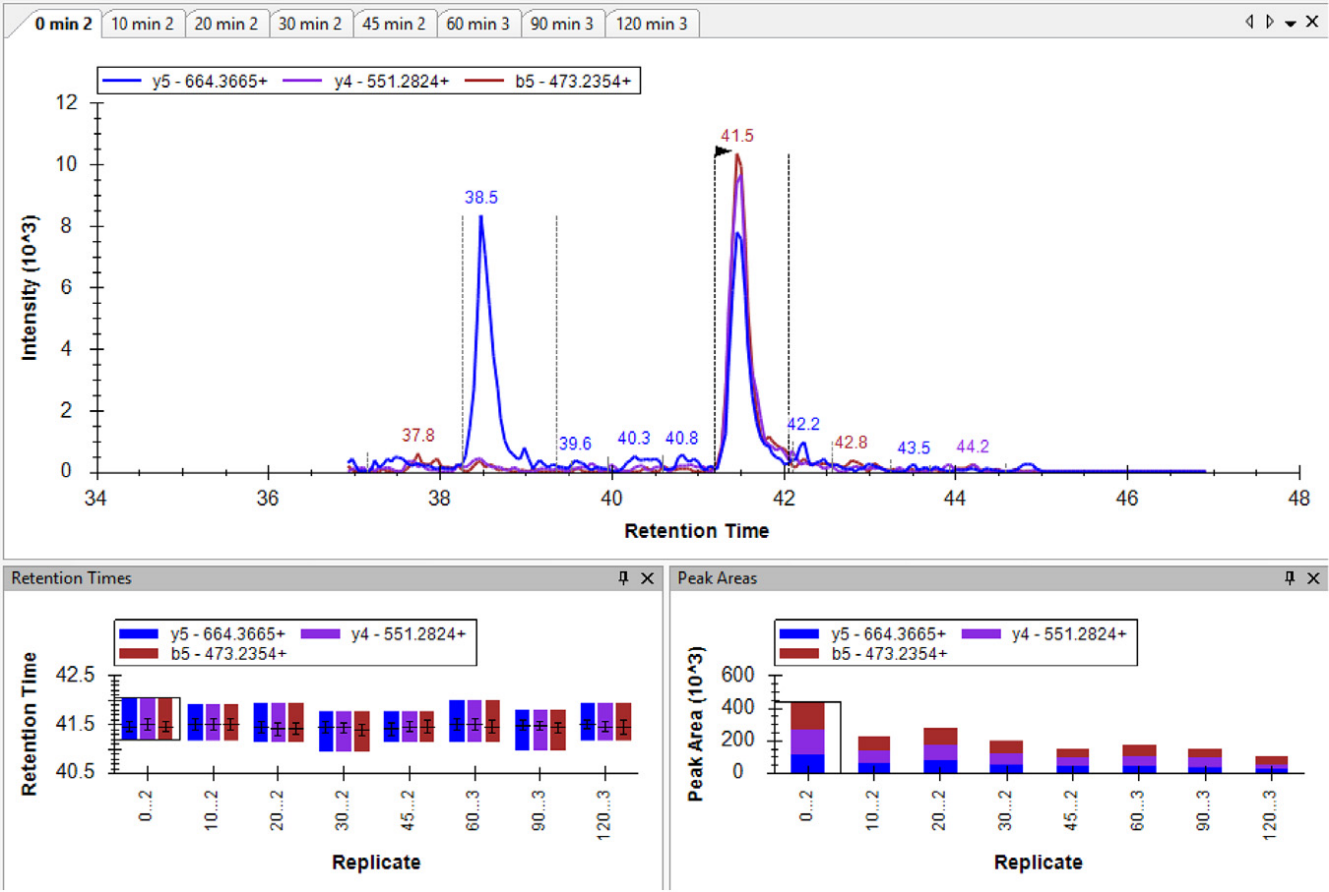
  **LC20-1P**
  **Figure:**
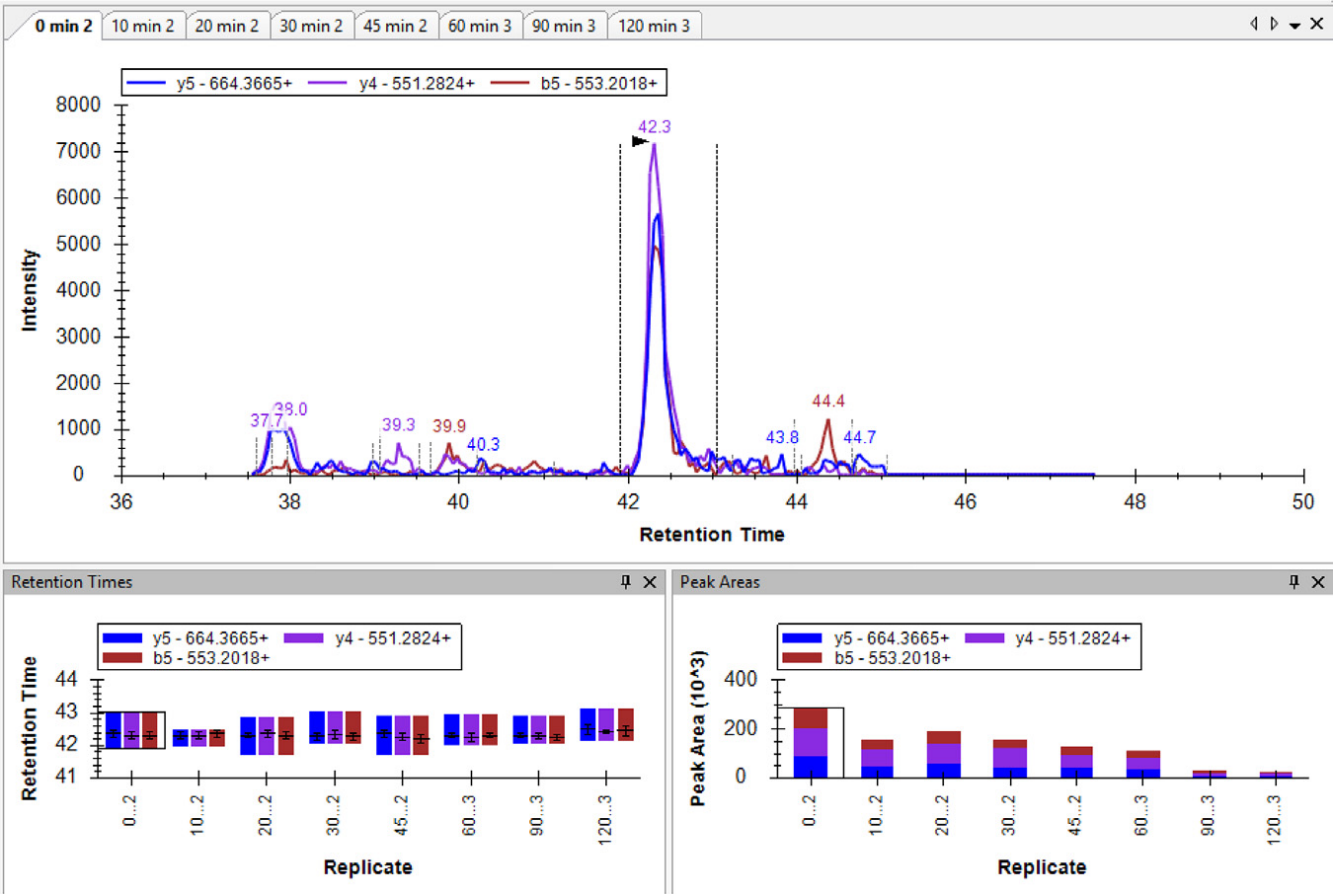
  **LC20-2P**
  **Figure:**
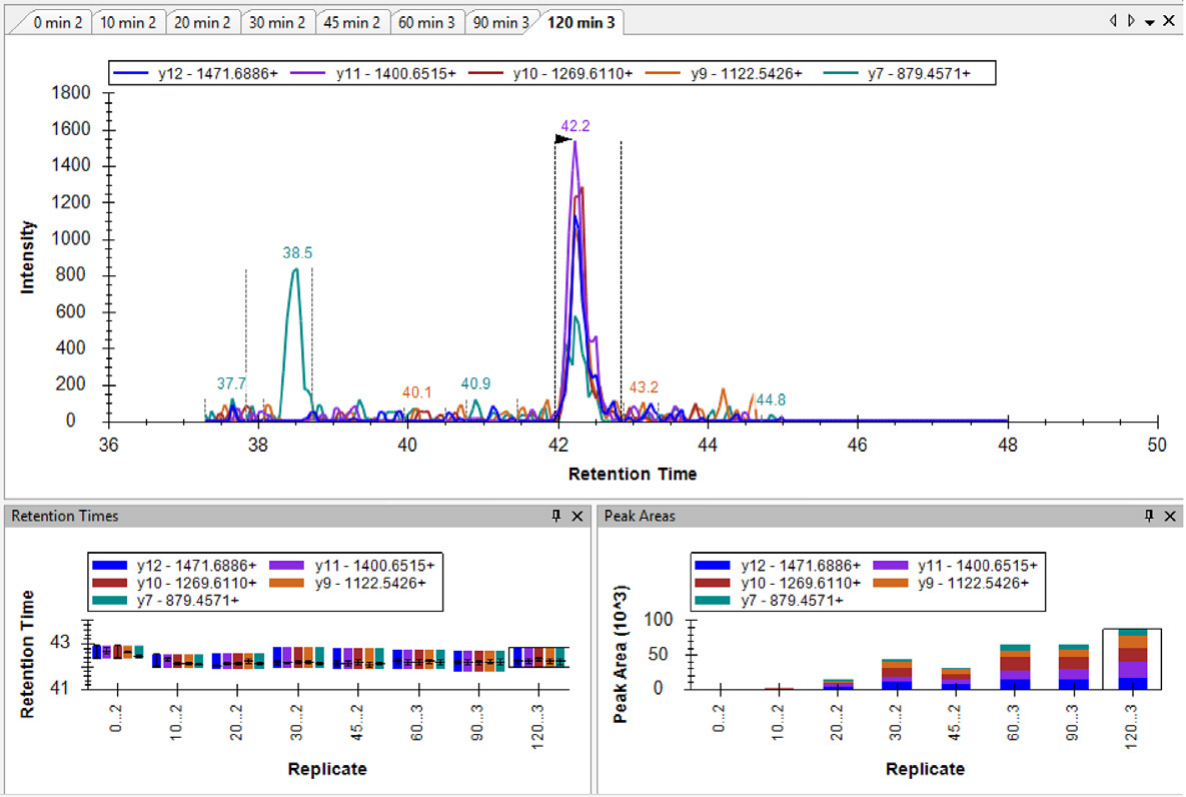
• The peak areas for the diagnostic transitions were obtained from the Skyline program and processed in Microsoft Excel. Summate the peak areas obtained for the three diagnostic transition ions used for each of the original precursor peptides.
  **Figure:**
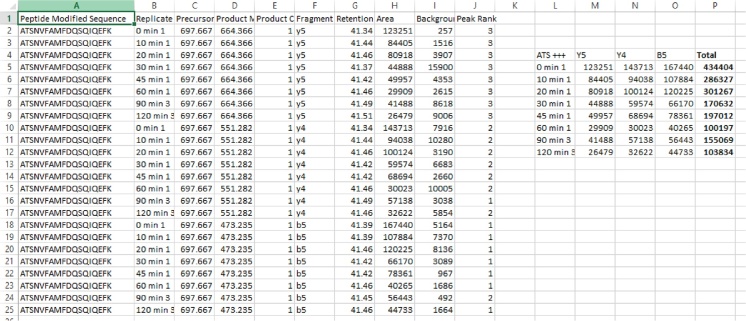
• The LC20 phosphorylation stoichiometry was calculated in Microsoft Excel using the equation provided in Section Calculation of LC20 phosphorylation stoichiometry.
  **Figure:**